# Association Between Contact Precautions and Transmission of Methicillin-Resistant *Staphylococcus aureus* in Veterans Affairs Hospitals

**DOI:** 10.1001/jamanetworkopen.2021.0971

**Published:** 2021-03-15

**Authors:** Karim Khader, Alun Thomas, Vanessa Stevens, Lindsay Visnovsky, McKenna Nevers, Damon Toth, Lindsay T. Keegan, Makoto Jones, Michael Rubin, Matthew H. Samore

**Affiliations:** 1 IDEAS Center of Innovation, Veterans Affairs Salt Lake City Health Care System, Salt Lake City, Utah; 2 Division of Epidemiology, Department of Internal Medicine, University of Utah School of Medicine, Salt Lake City

## Abstract

**Importance:**

The effectiveness and importance of contact precautions for endemic pathogens has long been debated, and their use has broad implications for infection control of other pathogens.

**Objective:**

To estimate the association between contact precautions and transmission of methicillin-resistant *Staphylococcus aureus* (MRSA) across US Department of Veterans Affairs (VA) hospitals.

**Design, Setting, and Participants:**

This retrospective cohort study used mathematical models applied to data from a population-based sample of adults hospitalized in 108 VA acute care hospitals for at least 24 hours from January 1, 2008, to December 31, 2017. Data were analyzed from May 2, 2019, to December 11, 2020.

**Exposures:**

A positive MRSA test result, presumed to indicate contact precautions use according to the VA MRSA Prevention Initiative.

**Main Outcomes and Measures:**

The main outcome was the association between contact precautions and MRSA transmission, defined as the relative transmissibility attributed to contact precautions. A contact precaution effect estimate (<1 indicates a reduction in transmission associated with contact precautions) was estimated for each hospital and then pooled over time and across hospitals using meta-regression.

**Results:**

In this cohort study of 108 VA hospitals, more than 2 million unique individuals had over 5.6 million admissions, of which 14.1% were presumed to have contact precautions with more than 8.4 million MRSA surveillance tests. Pooled estimates found associations between contact precautions and transmission to be stable from 2008 to 2017, with estimated transmission reductions ranging from 43% (95% credible interval [CrI], 38%-48%) to 51% (95% CrI, 46%-55%). Over the entire 10-year study period, contact precautions reduced transmission 47% (95% CrI, 45%-49%), and the intrafacility autocorrelation coefficient estimate was 0.99, suggesting consistent estimates over time within facilities. Larger facilities and those with higher admission screening compliance observed additional reductions in transmission associated with contact precautions (relative rate, 0.84; 95% CI, 0.74-0.96 and 0.74; 95% CI, 0.58-0.96, respectively) compared with smaller facilities and those with lower admission screening compliance. Facilities in the southern US had a smaller transmission reduction attributable to contact precautions (relative rate, 1.14; 95% CI, 1.01-1.28) compared with facilities in other regions in the US.

**Conclusions and Relevance:**

In this cohort study of adults in VA hospitals, transmissibility of MRSA was found to be reduced by approximately 50% among patients with contact precautions. These results provide an explanation for decreasing acquisition rates in VA hospitals since the MRSA Prevention Initiative.

## Key Points

**Question:**

Are contact precautions for pathogen transmission associated with reductions in person-to-person transmission of methicillin-resistant *Staphylococcus aureus* (MRSA) in US Veterans Affairs (VA) acute care hospitals?

**Findings:**

In this cohort study, transmission models were fit to data on 8.4 million surveillance tests from 5.6 million admissions to 108 VA hospitals between 2008 and 2017. The estimated reduction in transmissibility of MRSA associated with contact precautions was 47%.

**Meaning:**

In this large-scale study, contact precautions were associated with a 2-fold reduction in MRSA transmission, which suggests that the MRSA Prevention Initiative was associated with the decline in acquisition rates in VA hospitals.

## Teaser

This cohort study estimates the effectiveness of contact precautions on reducing methicillin-resistant *Staphylococcus aureus* (MRSA) transmission in US Department of Veterans Affairs hospitals based on the MRSA Prevention Initiative.

## Introduction

Antibiotic-resistant pathogens are a serious public health concern, and the association between the coronavirus disease 2019 pandemic and personal protective equipment supply availability has strained infection control programs across the country. While a number of approaches to prevent resistant organisms have been used,^1,2,3,4,5,6,7,8^ they are often implemented in bundles, which makes evaluating the individual components difficult. Given the role of contaminated health care workers in transmission of antibiotic-resistant pathogens, there is a theoretical basis for the use of contact precautions. However, an evidence base for the effectiveness of contact precautions has been slow to accumulate.

In 2003, the Society for Healthcare Epidemiology of America published guidelines^9^ recommending a 2-pronged approach: (1) active surveillance cultures to identify reservoirs of methicillin-resistant *Staphylococcus aureus* (MRSA) and vancomycin-resistant *Enterococcus* (VRE) and (2) contact precautions for patients identified as carriers. Subsequently, the Centers for Disease Control and Prevention (CDC) released updated guidelines recommending use of gloves and gowns when caring for all patients infected or previously identified as colonized with target antibiotic-resistant bacteria.^10^ In October 2007, the US Department of Veterans Affairs (VA) implemented a MRSA Prevention Initiative in all hospitals. The initiative included 4 elements: (1) universal surveillance for MRSA colonization; (2) contact precautions for patients identified as carriers of MRSA; (3) an emphasis on hand hygiene; and (4) an institutional culture change that placed responsibility for infection control on everyone with patient contact.^4^ Implementation of these guidelines, which have been recommended by the CDC as recently as July 2019,^11^ provides a natural context for assessing the effectiveness of contact precautions across a large and geographically diverse population of hospitals.

Previous studies of contact precautions used a variety of approaches but yielded limited results.^3,5,12,13,14,15^ The VA MRSA Prevention Initiative, later expanded to include nursing homes, has been credited in part with persistent decreases in MRSA infection rates since October 2007.^4,16,17,18,19^ However, contact precautions have become an increasing topic of debate, coincident with a number of factors including limited or inconsistent clinical trials evidence.^3,5^

To address this evidence gap, we estimated the effectiveness of contact precautions in reducing MRSA transmission in VA hospitals based on the MRSA Prevention Initiative. To our knowledge, this represents the largest such study, encompassing more than 100 acute care hospitals and 10 years of surveillance data. We developed models that incorporate the epidemiology of MRSA transmission to estimate the underlying transmission rate.

## Methods

### Overview

**Figure 1.**
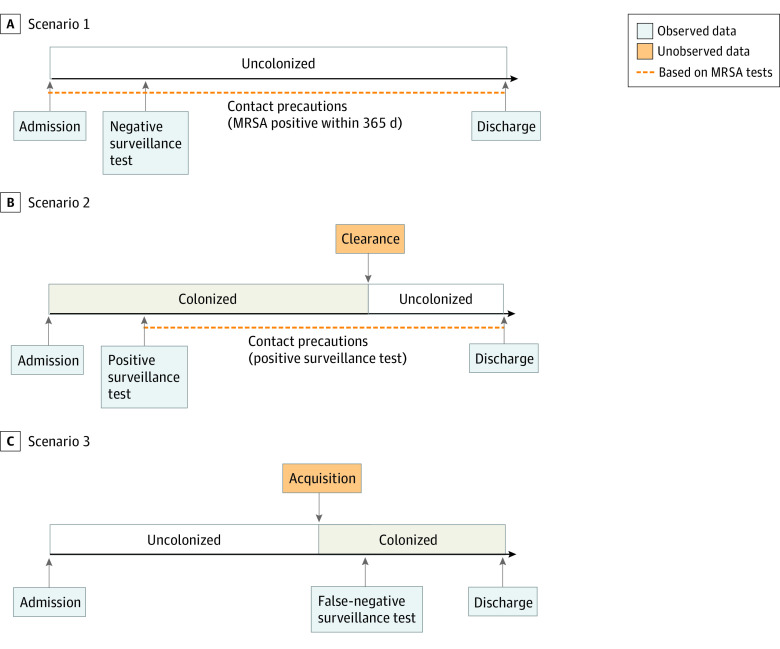
Transmission Model Illustration of the underlying transmission model, showing the possible transitions for patient colonization, and the relationship between the unobserved and observed data in the model in 3 scenarios. In (A), the patient remains uncolonized throughout the hospitalization but is assumed to have contact precautions due to a prior positive methicillin-resistant *Staphylococcus aureus* (MRSA) test result. Both (B) and (C) highlight the potential change in colonization status, with (B) illustrating that colonization does not always coincide with contact precautions and (C) illustrating the allowance for imperfect testing with a false-negative test result.

We fit a bayesian transmission model, extending previous work,^20,21^ to MRSA surveillance data to estimate important epidemiological parameters. Our transmission model incorporated 3 fundamental components (Figure 1): observed data, unobserved data, and model parameters. A summary of estimates of model parameters not described herein is available in the eTable in the Supplement. The unobserved data represent the underlying true timing of acquisition and clearance events, while accounting for imperfect surveillance test sensitivity, and are imputed based on the prescribed relationship with the observed data, represented by information we can obtain from the patients’ medical records. While it is not possible to perfectly observe patient colonization status during hospitalizations, the observed data are used to make inferences about the unobserved data using data augmentation,^22^ an approach for including unobserved data or latent variables. The combined observed and unobserved data are referred to as the augmented data. The study was reviewed and approved by the University of Utah Institutional Review Board and the Research and Development Committee of the VA Salt Lake City Health Care System, which waived patient consent because the project relied on retrospective analysis of existing patient records. The study followed the Strengthening the Reporting of Observational Studies in Epidemiology (STROBE) reporting guideline for cohort studies.

### Observed Data

We analyzed data from patients admitted to VA acute care hospitals from January 1, 2008, to December 31, 2017. Observed patient data included date; time and ward for each admission; transfer and discharge; times and results for MRSA surveillance tests; and times of MRSA-positive clinical cultures. Data were partitioned into five 2-year analysis periods to allow estimates of transmission to change over time and included all facilities with complete data spanning the 10-year study period. In data from 139 hospitals, 7 facilities were excluded because of missing MRSA surveillance data, 13 because of missing facility characteristics required for secondary analysis, and 11 because of incorrect surveillance results during the study period, leaving 108 facilities included in the final analysis.

### Unobserved Data

The true colonization status at admission, time of MRSA acquisition, and time of MRSA clearance for patients are considered unobserved data because they are unobservable. However, including the unobserved data in the model is required for accurate estimation of the epidemiological parameters.

### Model Parameters

We created a dynamic transmission model integrating clinical information with assumed mechanistic relationships, and specified using the model parameters. These included surveillance test sensitivity, importation probability, and transmission rate. An additional parameter was included to estimate the effectiveness of contact precautions, a measure of the relative change in transmissibility for patients with contact precautions compared with patients without contact precautions.

### Patient Flow

Patient ward location was tracked over time, which allowed the model to appropriately shift risk of transmission according to the movement of patients within the hospital. Our model also accounted for readmissions and incorporated dependencies between consecutive hospitalizations, including capturing contact precautions at subsequent admissions attributed to previous positive MRSA tests as prescribed by the MRSA Prevention Initiative.

### MRSA Importation

Patients colonized with MRSA at admission were considered an *importation*. Although patients may have had multiple admissions in the data, *importation probability* was defined as the probability that an individual was colonized with MRSA at the time of first admission. For patients with a previous hospitalization, their colonization status from the time of previous admission informed the probability of being colonized at the time of readmission. Between consecutive admissions, patients were assumed to both acquire and lose colonization at a constant rate. This assumption resulted in a simple formula for computing the probability of colonization at the time of readmission given the previous colonization status, the time since prior discharge, and the rates of acquisition and clearance between consecutive admissions (eMethods in the Supplement). For patients readmitted and colonized at their prior discharge, as the time since discharge increased, the probability that they remained colonized decreased because of the constant rate of clearance. The probability of colonization never reached 0 because there is a constant risk of community reacquisition between admissions.

### Transmission

Transmission was assumed to occur within wards, resulting in ward-specific transmission rates, and transmission between wards was assumed to be negligible. The underlying transmission model was the classic frequency-dependent model,^23^ where the force of infection, which is the rate at which susceptible individuals acquire an infectious pathogen, was assumed to be proportional to the colonization prevalence on the ward. The transmission rate parameter represents the proportional constant that describes the intensity of the force of infection. Because the transmission rate is distinct from colonization prevalence, it serves as a measure of transmission that is independent of prevalence. Given 2 wards with the same prevalence of MRSA, a higher transmission rate in 1 ward suggests an increased level of transmission.

### Contact Precautions

Contact precautions were modeled by defining 2 groups of patients who pose potentially distinct risks of transmission to other susceptible patients: those requiring contact precautions and those not requiring contact precautions, which is referred to as the baseline transmission rate. The VA MRSA Prevention Initiative guidelines were used to classify patients requiring or not requiring contact precautions, assuming that patients with a positive surveillance test result or clinical culture were appropriately recommended for contact precautions according to VA policy. This determination was based on the results of MRSA surveillance tests and clinical cultures. Accordingly, patients were given contact precautions 12 hours after a positive MRSA surveillance test, and 24 hours after a positive clinical culture for MRSA. The times from sample collection to initiating contact precautions were selected based on typical test turnaround time.^24^ Infectiousness of patients who required contact precautions was modeled with the contact precautions parameter, effectiveness of contact precautions, which represented the transmissibility of patients colonized with MRSA while receiving contact precautions relative to those not receiving contact precautions (eFigure 1 in the Supplement). This assumption implies that when estimated effectiveness of contact precautions is less than 1, contact precautions reduce transmission (contact precautions are effective) and when estimated effectiveness of contact precautions is greater than 1, contact precautions increase transmission.

### Clearance

Patients colonized with MRSA were assumed to clear MRSA colonization during their admission based on an average hospitalwide clearance rate. Once cleared, patients were assumed to immediately be at-risk to reacquire MRSA.

### Test Sensitivity

Similar to other studies,^14,25,26,27^ the assumption that there were no false-positive surveillance tests was made. Therefore, surveillance tests represented true negative, false negative, or true positive.

### Estimation

A bayesian framework using both the Gibbs sampler^28^ and the Metropolis-Hastings algorithm^29^ was used for parameter estimation. The bayesian model generated a distribution of parameter values, known as the posterior distribution, which formed the basis for the point estimates and credible intervals used in the secondary analysis. A broad background on bayesian models and the use of Markov Chain Monte Carlo can be found in Gilks et al.^30^

### Statistical Analysis

Posterior means and 95% credible intervals (95% CrI) for estimates of the effectiveness of contact precautions were computed separately for each facility, and estimates were pooled over the entire study period and for each 2-year time period. For the model across time, we assumed a heteroscedastic autoregressive covariance structure for facility dependence in time, allowing for differences in variance and estimates of temporal correlation for the effect of contact precautions.

In addition, multivariate meta-regression was used to identify facility-level characteristics associated with differences in the associations with contact precautions between facilities, along with 95% CIs. The facility-level characteristics considered in the meta-regression included facility size, census-defined regions and divisions, transmission rate, and estimates of compliance with the MRSA Prevention Initiative. These facility characteristics were considered to be statistically significant given a 2-sided *P* < .05. The Markov Chain Monte Carlo algorithm was implemented in C++ and all analyses were conducted on the VA Informatics and Computing Infrastructure (VINCI) platform. Data were analyzed from May 2, 2019, to December 11, 2020. The analysis of model posterior distributions used the *rmeta*,^31^ *metafor*,^32^ and *base*^33^ packages from The R Project for Statistical Computing. Additional technical details on the modeling assumptions and formula are available in the eMethods in the Supplement.

## Results

### Data Summary

This cohort study included 108 hospitals with more than 2 million unique individuals with more than 5.6 million admissions and more than 8.4 million MRSA surveillance tests (9.3% positive). Among all admissions, 14.1% (n = 794 814) required contact precautions sometime during their stay based on a positive test result for MRSA. Of those, 58% were initiated at the time of admission owing to a positive test result of MRSA within the previous 365 days, 29% were initiated following a positive surveillance test result within 24 hours of admission, and 13% were initiated after a positive follow-up test result that occurred after a negative admission test result.

### Contact Precautions Over Time

**Figure 2.**
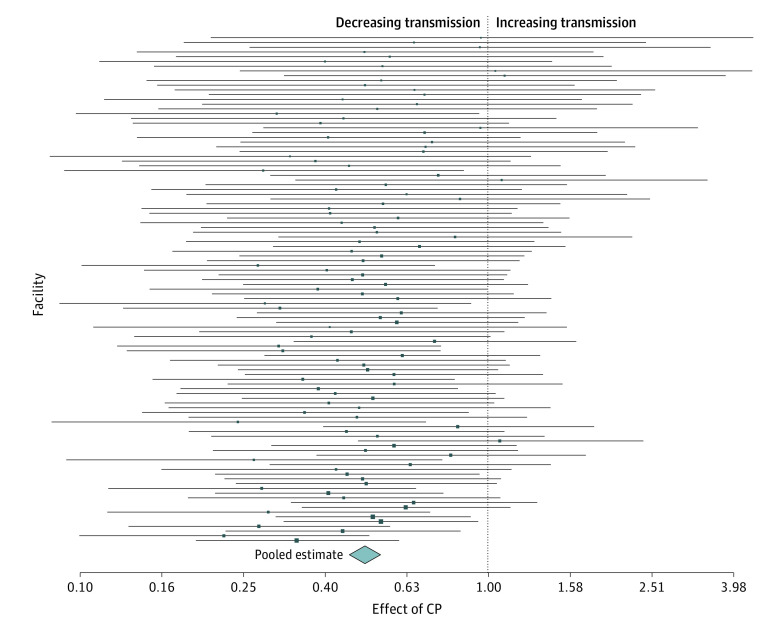
Facility-Specific Estimates From 2012 to 2013 Forest plot showing the facility-specific estimates represented by squares, along with the corresponding 95% CIs for each of the facilities during the 2 years from 2012 to 2013. The pooled estimate is represented by the diamond at the bottom, with the width of the diamond indicating the 95% CI for the pooled estimate. The size of the squares reflects the precision of the facility-specific estimates. CP indicates contact precautions.

**Figure 3.**
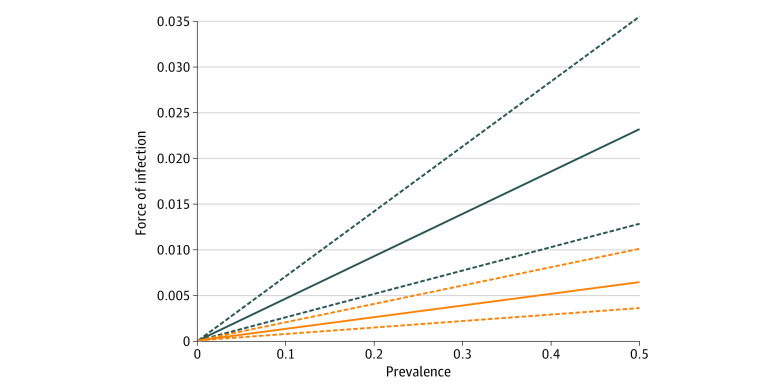
Association Between Prevalence and Force of Infection Illustration of the association between ward prevalence and the estimated force of infection in a single general acute medicine ward contrasting the differential in the transmissibility of patients not with contact precautions (blue) with those with contact precautions (orange). The dashed lines represent the 95% CIs.

Pooled estimates of the effectiveness of contact precautions varied during the period from January 1, 2008, to December 31, 2017, with estimates ranging from 0.49 (95% CI, 0.45-0.54) to 0.57 (95% CI, 0.52-0.62). During a 2-year period from 2012 to 2013, the estimates varied by facility (Figure 2), with 96.3% of facility estimates indicating a reduction in transmission and 22.2% of facility 95% CIs below 1, providing evidence for reduced transmission owing to contact precautions. Figure 3 illustrates the association between the estimates of effectiveness of contact precautions and the force of infection in a single hospital ward during this same period. Pooling the estimates across all facilities during this time period resulted in an estimated reduction in the transmission rate by a factor of 0.50 (95% CI, 0.45-0.54). Similar results were obtained during each of the time periods (eFigures 2-5 in the Supplement).

### Overall

The meta-analysis pooled over the entire study period gave an estimated reduction in transmission for patients who required contact precautions by a factor of 0.53 (95% CI, 0.51-0.55). The test for residual heterogeneity in the random-effects model produced an estimated variance of 0.0051 (*P* > .99), suggesting no evidence for heterogeneity among the facilities, and the autocorrelation coefficient for the intrafacility estimates of the effectiveness of contact precautions was 0.99, suggesting consistent estimates over time within facilities.

### Pooled Estimates With Moderators

**Figure 4.**
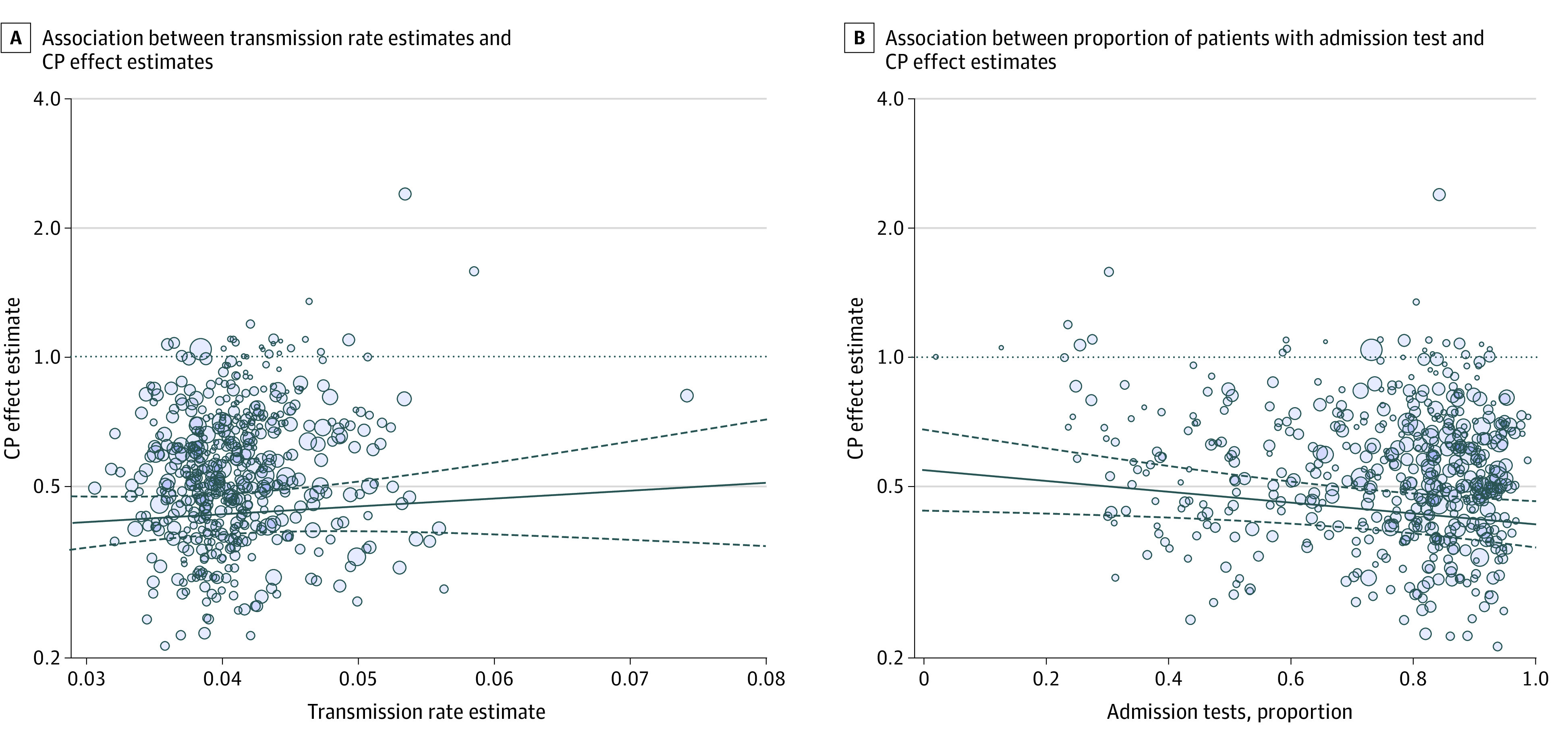
Association Between Facility Measures and Estimated Effectiveness of Contact Precautions Bubble plot showing the association between the transmission rate estimates and the contact precautions (CP) effect estimate (A). The lines show the association between the estimated CP effect parameter and the transmission rate (solid line) and the 95% CIs (dashed lines), and the dotted line represents no effect. The blue circles are the estimates for each facility with the size of the dots reflecting the precision. Larger dots reflect low variance in the estimates. There is a slight increasing association between transmission rate and CP effect. On the right (B) is a bubble plot showing the association between contact precautions effect and proportion of admissions having an admission test. A decreasing association between the proportion of admission tests and the contact precautions effect parameter is shown.

Facility characteristics associated with estimates of effectiveness of contact precautions are presented in the Table. In particular, moderate and large facilities (having a mean daily census greater than 55.5 patients and 77.7 patients per day, respectively) have additional reductions in transmission associated with contact precautions (relative rate, 0.81; 95% CI, 0.71-0.93 and 0.84; 95% CI, 0.74-0.96, respectively) compared with small facilities (reference), and facilities in the South tend to have a smaller reduction in transmission than in other parts of the country (relative rate, 1.14; 95% CI, 1.01-1.28). Facilities with a higher proportion of admissions having an admission test were associated with a reduced transmissibility attributed to contact precautions (relative rate, 0.74; 95% CI, 0.58-0.96), but there is no evidence that a facility’s baseline transmission rate is associated with the estimated effectiveness of contact precautions. These associations between estimated effectiveness of contact precautions and the baseline transmission rate (Figure 4A), and the proportion of admissions with an admission test (Figure 4B), are illustrated across a range of values. The proportion of patient-days with contact precautions was not associated with the estimated effectiveness of contact precautions.

**Table.** Multivariable Model Evaluating Associations Between Facility Characteristics and Facility-Specific Estimates of the Effect of Contact Precautions on Transmission

Variable	Relative rate (95% CI)	*P* value	No. (%) (N = 108)
Transmission rate^a^	67.96 (0.02-232 474.4)	.31	NA
Proportion admission tests^b^	0.74 (0.58-0.96)	.02	NA
Proportion CP days^c^	1.29 (0.46-3.58)	.63	NA
Rural location	1.12 (0.94-1.33)	.22	12 (11)
Region			
Northwest	1 [Reference]	NA	21 (20)
Midwest	0.92 (0.81-1.04)	.17	31 (29)
South	1.14 (1.01-1.28)	.04	32 (31)
West	1.05 (0.92-1.20)	.44	23 (20)
Mean daily census			
0-22.7	1 [Reference]	NA	27 (25)
22.8-55.4	0.90 (0.79-1.04)	.15	27 (25)
55.5-77.6	0.81 (0.71-0.93)	.003	27 (25)
77.7-194	0.84 (0.74-0.96)	.01	26 (25)

Abbreviations: CP, contact precautions; NA, not applicable.

^a^ Facility-specific transmission rate obtained by pooling across all wards within each facility.

^b^ The proportion of admissions that have an admission surveillance test (ie, within 24 hours of admission).

^c^ The proportion of total patient-days that include contact precautions.

## Discussion

We report the results of a large study of the estimated effectiveness of contact precautions in the prevention of patient-to-patient transmission of MRSA in VA hospitals. Fitting models of transmission, we estimate that contact precautions were associated with a reduction in transmission by 47%. This finding may explain why rates of MRSA acquisition have decreased in VA hospitals since implementation of the MRSA initiative.

We applied statistical methods similar to those used previously in smaller studies,^13,14,15^ but adapted them to allow for hospitalwide analysis that accounts for transmission within wards. We increased statistical power by estimating the effectiveness of contact precautions for each individual hospital and then generating a pooled estimate of the overall association using meta-regression techniques. Our study suggests that analyzing data from a single institution has limited statistical power to estimate the association with contact precautions, even when the results of surveillance tests are accumulated over many years.

The effectiveness of contact precautions likely depends on health care personnel’s adherence to recommended hand hygiene and personal protective equipment practices. Because infection control practices are not measured consistently across VA hospitals, it was not possible to directly evaluate this association. However, findings suggest that a possible surrogate of adherence to infection control protocols for MRSA, namely, the proportion of hospitalized patients who were tested for MRSA on admission, was associated with increased benefit of contact precautions.

The estimated effectiveness of contact precautions is a ratio between 2 fitted slopes, one which relates the per capita transmission rate to the prevalence of MRSA colonization in patients who are not receiving contact precautions and the other which relates the per capita transmission rate to the prevalence of MRSA colonization in patients who receive contact precautions. Conceivably, a hospital with extremely high adherence to hand hygiene and body substance isolation could have sufficiently low transmission of MRSA from patients not requiring contact precautions to make it difficult to detect a difference relative to patients requiring contact precautions. Our results suggest that this scenario is not a frequent occurrence. Rather, we found no evidence of an association between the transmission rate while not requiring contact precautions and the estimated effectiveness of contact precautions.

Prior studies have suggested that the MRSA Prevention Initiative resulted in a sustained reduction in health care–associated MRSA infections in VA hospitals.^4,16,17,19,34^ A previous study reported that in addition to a reduction of the MRSA infection rate, the MRSA transmission rate across a large number of VA hospitals declined following the MRSA Prevention Initiative.^21^ However, these studies did not directly measure the association between contact precautions and any elements of the bundle on MRSA transmission.

Most days that patients colonized with MRSA are not receiving contact precautions are contributed by patients who acquire MRSA during their hospitalization because they have a longer delay in detection. Detection of patients who import MRSA is more prompt because of the high level of adherence to admission screening. False-negative MRSA surveillance tests also contribute to not determining whether patients with MRSA colonization, either acquired or imported, require precautions. Further work is warranted to explore the implications of different screening policies for the estimation of the effectiveness of contact precautions.

The ongoing controversy over contact precautions arises from the absence of evidence from randomized clinical trials and contradictory results among existing studies.^35,36^ Many previous studies were limited by methodologic issues, such as before-and-after study designs, concurrent changes in infection control, poor outcome measures, small sample sizes, short study duration, or single-center studies. Here, we present the results of a rigorous multicenter observational study using the largest source of MRSA surveillance data in the US.

### Limitations

This study has limitations. This study had the potential for some patients to be misclassified with respect to whether they required contact precautions and when. There may have been delays in instituting precautions for patients who had positive surveillance tests or clinical cultures for MRSA; in some cases, contact precautions may have been omitted altogether. In contrast, a positive test result for MRSA is not the only reason for a patient requiring contact precautions. Some patients classified as not requiring contact precautions may have received contact precautions for a reason other than documented MRSA colonization or infection. However, both types of misclassification would bias the estimate of the effectiveness of contact precautions toward the null. Another study limitation is that patient-level factors such as use of therapeutic agents, which may be more common among patients testing positive for MRSA and may act as confounders, were not included in the analysis.

## Conclusions

In this cohort study, we observed a reduction in transmission of approximately half, associated with the VA policy of contact precautions for MRSA. At the facility level, there was variability in the estimates, and we found that increased effectiveness of contact precautions was associated with moderate to large facilities. Facilities having higher compliance with admission screening were associated with additional reductions in transmission associated with contact precautions. Further work is needed to better understand and explain the variation, and to incorporate cost-effectiveness analysis to provide optimal guidance on contact precautions implementation.

## Notes

Supplement. **eMethods.** **eFigure 1.** Relative Rate of Transmissibility Associated With Contact Precautions **eFigure 2.** Forest Plot for Transmissibility Associated With Contact Precautions 2008-2009 **eFigure 3.** Forest Plot for Transmissibility Associated With Contact Precautions 2010-2011 **eFigure 4.** Forest Plot for Transmissibility Associated With Contact Precautions 2014-2015 **eFigure 5.** Forest Plot for Transmissibility Associated With Contact Precautions 2016-2017 **eTable.** Summary of Transmission Model Parameter Estimates **eReferences.**
