# Supplementary material for: Association Between Contact Precautions and Transmission of Methicillin-Resistant Staphylococcus aureus in Veterans Affairs Hospitals
Source: JAMA Netw Open. 2021 Mar 15;4(3):e210971. doi: 10.1001/jamanetworkopen.2021.0971 (PMC7961311; doi:10.1001/jamanetworkopen.2021.0971)
Supplement: Supplement. — eMethods. eFigure 1. Relative Rate of Transmissibility Associated With Contact Precautions eFigure 2. Forest Plot for Transmissibility Associated With Contact Precautions 2008-2009 eFigure 3. Forest Plot for Transmissibility Associated With Contact Precautions 2010-2011 eFigure 4. Forest Plot for Transmissibility Associated With Contact Precautions 2014-2015 eFigure 5. Forest Plot for Transmissibility Associated With Contact Precautions 2016-2017 eTable. Summary of Transmission Model Parameter Estimates eReferences. [file jamanetwopen-e210971-s001.pdf]

Supplemental Online Content

Khader K, Thomas A, Stevens V, et al. Association between contact precautions and transmission of methicillin-resistant *Staphylococcus aureus* in Veterans Affairs hospitals. *JAMA Netw Open*. 2021;4(3):e210971. doi:10.1001/jamanetworkopen.2021.0971

eMethods.

eFigure 1. Relative Rate of Transmissibility Associated With Contact Precautions

eFigure 2. Forest Plot for Transmissibility Associated With Contact Precautions 2008-2009

eFigure 3. Forest Plot for Transmissibility Associated With Contact Precautions 2010-2011

eFigure 4. Forest Plot for Transmissibility Associated With Contact Precautions 2014-2015

eFigure 5. Forest Plot for Transmissibility Associated With Contact Precautions 2016-2017

eTable. Summary of Transmission Model Parameter Estimates

eReferences.

This supplemental material has been provided by the authors to give readers additional information about their work.

© 2021 Khader K et al. *JAMA Network Open*.

Overview

This supplementary document describes in more detail, the underlying methods that were implemented for the Bayesian transmission model analysis, including the underlying formula, description of parameters and their assumptions, and Bayesian estimation features in our algorithm including prior distributions and steps for updating the augmented data. Additionally, we have included some results complementary to those in the main manuscript, but which were not of primary interest.

The figures and tables found in this supplementary document are listed below.

	Name
eFigure 1	Relative Rate of transmissibility associated with contact precautions
eFigure 2	Forest plot for transmissibility associated with contact precautions 2008 – 2009
eFigure 3	Forest plot for transmissibility associated with contact precautions 2010 – 2011
eFigure 4	Forest plot for transmissibility associated with contact precautions 2012 – 2013
eFigure 5	Forest plot for transmissibility associated with contact precautions 2014 – 2015
eFigure 6	Forest plot for transmissibility associated with contact precautions 2016 – 2017
eTable 1	Summary of transmission model parameter estimates

eMethods

Bayesian Model

The dynamic transmission model that we implemented for this analysis is an extension of the general modeling framework that we have described previously,[1–3] which is represented as a Susceptible-Infectious-Susceptible (SIS) model. In this implementation, we are principally interested in investigating the association between contact precautions and transmission of MRSA across the VA. The model was implemented within a Bayesian framework, depending on a dataset D, which consists of observed data and is also augmented with unobserved data. Examples of the unobserved data are the times of colonization and clearance, specifying the underlying colonization dynamics. We assume that the list of events in the data are ordered by time, and model parameters are represented by θ. We can express the likelihood for the augmented data D, given the model parameters θ as

$$\pi(D \mid \theta) = \prod_{\{e \in D\}} g(e, t_e;\ \theta) h(t_e, t_{e^-}; \theta).$$

Here, we define t_e and t_{e^-} as the times of the events e and the prior event, e^-. $g(e, t_e;\ \theta)$ is a function that returns event-specific contributions to the likelihood, and varies according to the type of event e (described in more detail below), while $h(t_e, t_{e^-}; \theta)$ gives the probability that no event is observed between times t_{e^-} and t_e.

Transmission

We assume that acquisitions at time t for a given uncolonized individual occur according to the frequency-dependent model of transmission, at a rate $\lambda(t) \cdot C(t)/N(t)$. In principal, we could allow $\lambda(t)$ to take on a number of functional forms, but for our purposes we assume the transmission rate to be constant in time. Additionally, we assume that transmission occurs within wards and not across

© 2021 Khader K et al. *JAMA Network Open*.

wards. So for a given facility, assuming that there are N_W wards, we assume there are ward-specific transmission rates $\lambda_1, \lambda_2, \dots, \lambda_{N_W}$. Combining the ward-specific transmission rates with relative rate of transmission associated with for contact precautions, $\mathrm{CP_e}$, we have that for each ward i, acquisitions at time t for each uncolonized invidual occur at a rate

$$g(e;\theta) = \lambda_i \cdot \frac{C_i^0(t)}{N_i(t)} + \mathrm{CP_e} \cdot \lambda_i \cdot \frac{C_i^\kappa(t)}{N_i(t)}$$

where $C_i^0(t), C_i^\kappa(t)$ and $N_i(t)$ are the number of colonized not on contact precautions, number of colonized and on contact precautions and total number of patients respectively on ward i at time t.

Given this framework for transmission, we can now define the term $h(t_e, t_{e^-}; \theta)$ which gives the probability that no events occur between the consecutive events e^- and e, and is given by the formula

$$h(t_e, t_e;\ \theta) = \exp\left\{-S(t_e-)\int_{t_e-}^{t_e} \sum_{i=1}^{N_W} \lambda_i \cdot \frac{C_i^0(t_e-)}{N_i(t_e-)} + \mathrm{CP_e} \cdot \lambda_i \cdot \frac{C_i^\kappa(t_e-)}{N_i(t_e-)} \ - C(t_e-)\eta dt\right\},$$

where $S(t)$ is the number of susceptible patients at time t and η is the rate of loss of colonization for patients.

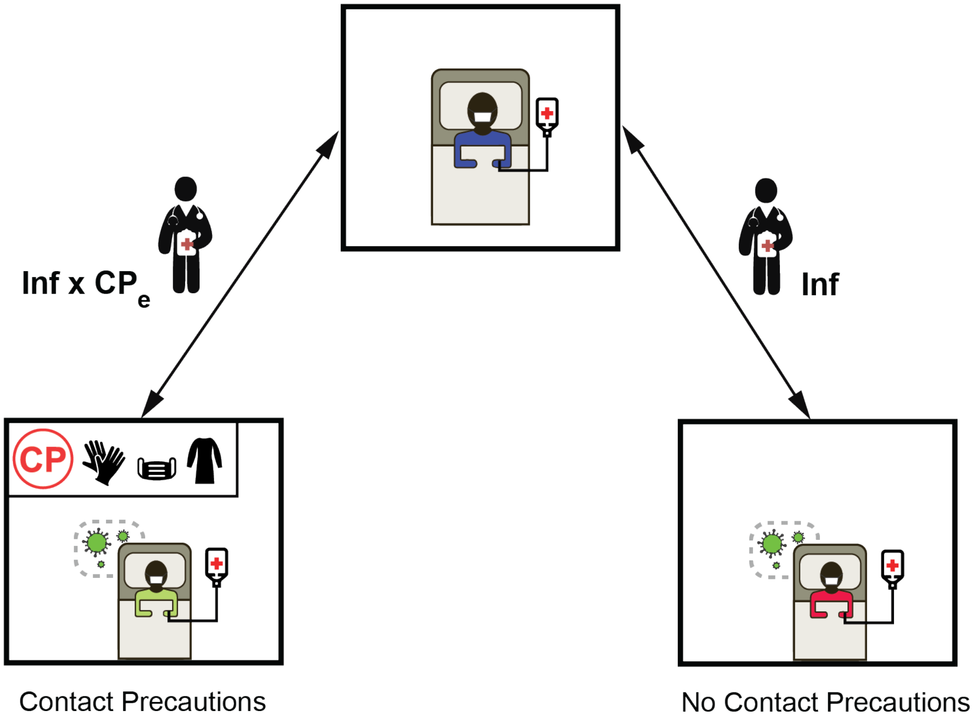

eFigure 1: Illustration of the relationship between the baseline transmission rate (**Inf**) and the relative rate of transmission associated with contact precautions (**CP$_e$**), which acts as a multiplier of the baseline transmission rate. The patient on the right is not on precautions while the patient on the left is on contact precautions. The transmission rate, assummed to be mediated through heatlhcare workers is modified appropiately.

© 2021 Khader K et al. *JAMA Network Open*.

Clearance
Although in reality, there are a variety of factors that influence clearance of MRSA, for simplicity we assume that for a given colonized patient, they will lose colonization at a constant rate η. Thus, this parameter provides an overall average estimate of the rate of decolonization across all wards and all factors that contribute to clearance. In our model, when a patient loses colonization, the corresponding event contributes $g(e;\ \theta) = \eta$ to the likelihood.

Surveillance tests
Surveillance tests in this analysis are all nasal swabs, yet there is some variation across the VA in testing methodologies, and those have changed over time. We have simplified our model by assuming a single set of surveillance test parameters, yet these are fit at the facility level and can change in each two-year period of analysis. We assume that a negative test results can represent either a true negative or a false negative, which depends on the unobserved colonization status reflected in the augmented data. We define $s(e)$ to be an indicator variable representing the fact that the patient corresponding with event e is colonized at time t_e. Given this notation, the formula for the probability $g(e;\ \theta)$ of the negative test is given by $\phi^{s(e)}$ and the probability of a positive test is $1 - \phi^{s(e)}$, where ϕ is the probability of a false negative test. Additionally, we assume that there are no false positives in the data.

Importation (we assume a single importation probability for the facility)
The status of a patient at the time of admission contributes to the likelihood based on their probability of being colonized, or importing to the facility at the time of admission. This importation probability depends on whether the admission event is the first admission or a readmission. Readmissions are modeled by a continuous time Markov chain in order to account for the possibility of switching colonization status during the period between consecutive admissions. In particular, between consecutive admissions, uncolonized individuals become colonized at rate α and colonized lose colonization at rate γ. From these two parameters, we define the "steady-state", or first-admission importation probability as $\nu = \alpha/(\alpha + \gamma)$. Therefore, for a first admission event, we assume a constant importation probability ν into the facility so that the contribution to the likelihood is

$$g(e;\theta) = \begin{cases} \nu, & s(e) = 1 \\ 1 - \nu, & s(e) = 0 \end{cases}$$

For a patient whose admission event e is a readmission, their importation probability is computed using information from their previous discharge e_d. In particular, we use standard methods for computing transition probabilities of a homogeneous CTMC during the period of time from t_d to t_e. It is straighforward to compute the probability of importation at a readmission, and the contribution to the likelihood is given by

$$g(e;\ \theta) = \begin{cases} \nu\left[1 - \exp\left\{-\Delta t \dfrac{\gamma}{1-\nu}\right\}\right], & s(e) = 1, s(e_d) = 0 \\ \nu + (1-\nu)\left[\exp\left\{-\Delta t \dfrac{\gamma}{1-\nu}\right\}\right], & s(e) = 1, s(e_d) = 1 \\ 1 - \nu + \nu \exp\left\{-\Delta t \dfrac{\gamma}{1-\nu}\right\}, & s(e) = 0, s(e_d) = 0 \\ (1-\nu)\left[1 - \exp\left\{-\Delta t \dfrac{\gamma}{1-\nu}\right\}\right], & s(e) = 0, s(e_d) = 1 \end{cases}$$

© 2021 Khader K et al. *JAMA Network Open*.

where $\Delta t = t_e - t_{e_d}$.

In-situ probability
At the time of the beginning of the study, there will be patients present in the facility that were admitted prior to the beginning of the study. For those individuals, their probability of colonization at the beginning of the study is treated as a nuisance parameter, σ, called the *in-situ* probability. For a patient present at the onset of the study, their first observation which indicates presence contributes $g(e;\ \theta) = \sigma^{s(e)}(1-\sigma)^{1-s(e)}$ to the likelihood.

Non-random events
For all other events e assumed to be observed without error (e.g. admission), $g(e;\theta) = 1$.

Prior Distribution
Below we summarize the prior distributions that help to complete the posterior distribution. We use a gamma distribution as the prior distribution for outpatient decolonization rate γ and for inpatient decolonization rates η. The in-situ probability (σ) and false negative probability (ϕ) are given uninformative Beta priors. The first-admission importation probability (ν) has log-normal distribution for prior while the log-transmission rate parameter (λ) and the log of the relative rate of transmission associated with contact precautions ($\mathrm{CP_e}$) have normal priors.

MCMC Updates
Now we describe the sequence of steps used to update both the augmented data and the model parameters. Estimation of parameters was performed using Markov chain Monte Carlo (MCMC), an iterative algorithm for generating parameter distributions. Estimation within each iteration of the MCMC algorithm involved generating a new sample of both the augmented data and the parameters. Given the observed data and the current parameter values, new augmented data (or patient histories) consistent with the observed data and the parameter values were proposed, and accepted, with a probability that depended on the relative likelihood of the models with the proposed and current augmented data. If the proposed augmented data were not accepted, then the current augmented data would remain until the next iteration through the MCMC. Given an updated augmented data, parameter values were proposed using both Gibbs sampler,[4] and the Metropolis-Hastings algorithm,[5] based on the new augmented data. This process of updating the augmented data and parameter values was iterated, and resulted in a collection of parameter values, having a distribution consistent with the likelihood, conditioned on all observed and unobserved data. This collection of parameter values, known as the posterior distribution for the parameters, formed the basis for the point estimates and credible intervals.

Augmented data
We use stochastic integration to account for the uncertainty in the augmented data set by updating the augmented data by sampling from the conditional distribution of each patient's history of colonization given the observed data, the current parameter values, and the current history of all other patients.

© 2021 Khader K et al. *JAMA Network Open*.

To sample the augmented data, we sequentially step through each patient admission and propose a new sequence of times that the patient might become colonized and decolonized. This proposal depends on the probability that the patient is an importation at the time of admission, as well as the current colonization and decolonization rates. The newly proposed augmented data is then accepted according to acceptance probability, following the Metropolis sampling scheme.

Patient Histories

Patient histories are updated sequentially conditional on all parameters, all other patient states and the admission, discharge and surveillance test data for the given patient. Roughly speaking this is accomplished by simulating an appropriate continuous-time Markov chain and then accepting or rejecting the proposed simulated history using a Metropolis-Hastings update. This approach benefits from having a relatively low rejection rate, although it is computationally intensive. Nonetheless, mixing of the overall chain converges rapidly.

Updating Patient History

For a given patient, we order and label all events that influence the patients colonization state during their history represented in the data. These events have corresponding times $t_0, t_1, \dots, t_n$ and include surveillance tests and changes in the number of other colonized and susceptible patients through admission, discharge, acquisition and clearance. Given the sequence of events, we define the sequence of transition matrices $Q_1, Q_2, \dots, Q_n$ and state probabilities $P_0, P_1, \dots, P_n$ where the patient states are represented by $X_0, X_1, \dots, X_n$. The transition matrices define the transition probability for the state variable between two consecutive events, that is Q_i determines the transition probability between X_{i-1} and X_i. Updating the patient history occurs in two steps. First, we sample from the discrete-time embedded Markov chain to generate candidate states and then we obtain the continuous extension for the patient that incorporates the times of the patient state changes. In the first step, we generate the states X_i from the full distribution $P(X_0, X_1, \dots, X_n) \propto P_0(X_0) \prod_{\{i=1\}}^{n} P_i(X_i) Q_i(X_{i-1}, X_i)$. After that, we iterate through the following algorithm to determine whether we accept or reject the sample. For $i = 1, \dots, n$, we sample $Y_i | X_{i-1}, Q_i$ until time t_i. If $Y_i(t_i) = X_i$, we accept the value, otherwise we reject it.

Transmission parameter updates

To update the log-transmission rate parameter, λ, and the log of the relative rate of transmission associated with contact precautions, $\mathrm{CP_e}$, we use Metropolis sampling. For each of the two parameters, we propose a new parameter value by adding a standard normal random variable with small variance to the current parameter value. The proposed sample is accepted or rejected according to the standard Metropolis sampling scheme. To improve mixing, this process was iterated 100 times, keeping the 100th update for the new parameter value.

Inpatient clearance update

To update the inpatient decolonization rate, η, we use Gibbs sampling. We generate a $\mathrm{Gamma}(a, b)$ random variable where a gives the total number of decolonization events, and b gives the total patient time at risk for decolonization according to the augmented data.

Outpatient parameter updates

© 2021 Khader K et al. *JAMA Network Open*.

To update the outpatient colonization rate α and decolonization rate γ, we use Metropolis sampling. For each of the two parameters, we propose a new parameter value by adding a standard normal random variable with small variance to the current parameter value. The proposed sample is accepted or rejected according to the standard Metropolis sampling scheme. To improve mixing, this process was iterated 10 times, keeping the 10th update for the new parameter value.

False negative update
To update the value of the false negative parameter ϕ, we use Gibbs sampling, generating a $\mathrm{Beta}(a, b)$ random variable where a is the number of false negative tests based on the augmented data, and b is the total number of tests in the dataset.

In-situ update
To update the in-situ probability of being colonized, σ, we use Gibbs sampling, generating a $\mathrm{Beta}(a, b)$ random variable where a gives the total number of individuals present at the beginning of the study who are colonized according to the augmented data, and b is the total number of individuals present at the beginning of the study.

In eFigures 1-5 (or eFigures 2-6), we include Forest plots that illustrate the estimates of the relative rate of transmission associated with contact precautions for each of the two-year periods analyzed in this study. The Forest plots provide the estimate and 95% confidence interval for each facility included in the study, and the pooled estimate for that same time period, represented by the diamond.

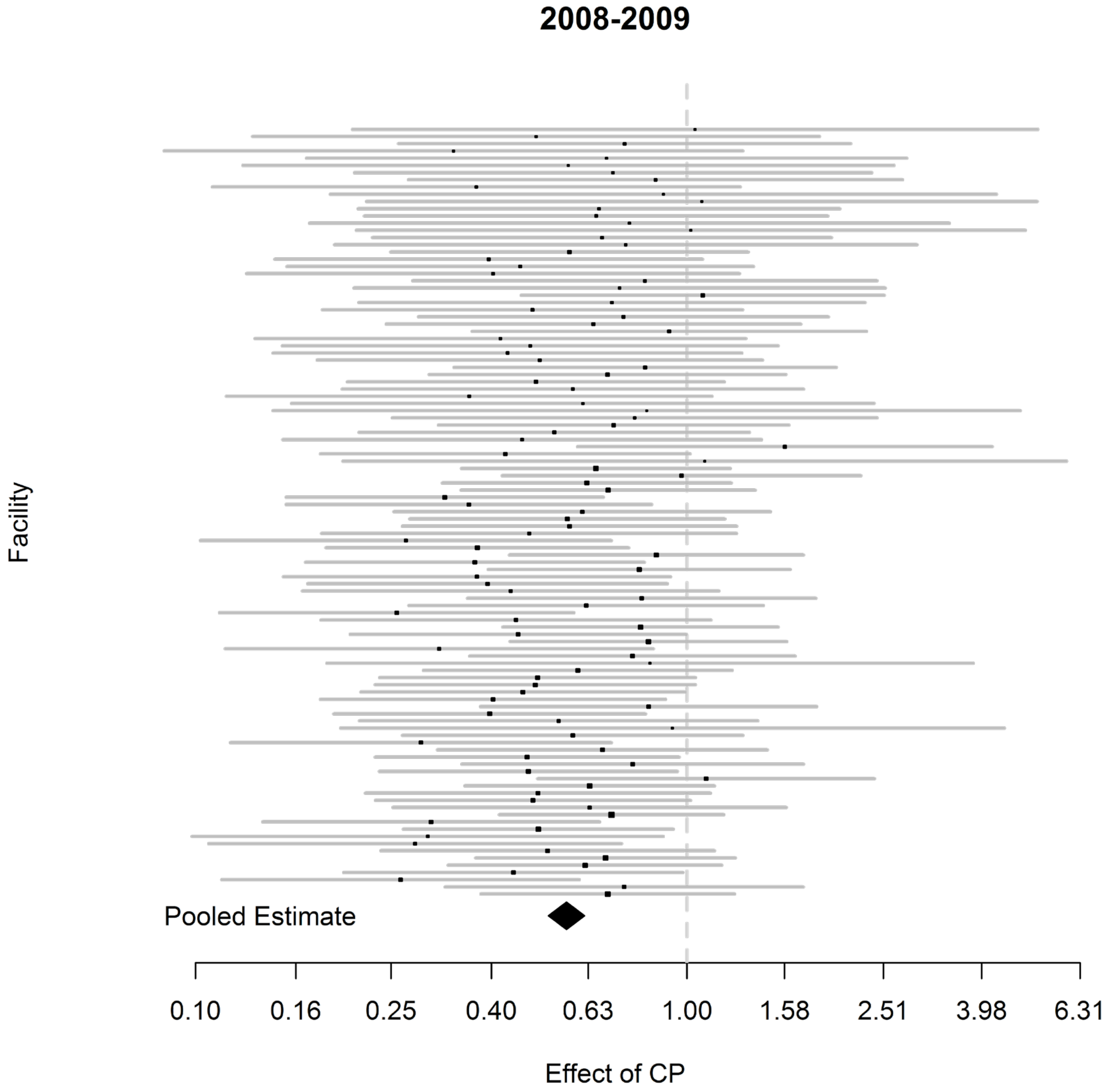

eFigure 2: Forest plot showing the facility-specific estimates along with 95% confidence intervals for each of the facilities during the two years from 2008-2009, and the pooled estimate represented at the bottom.

© 2021 Khader K et al. *JAMA Network Open*.

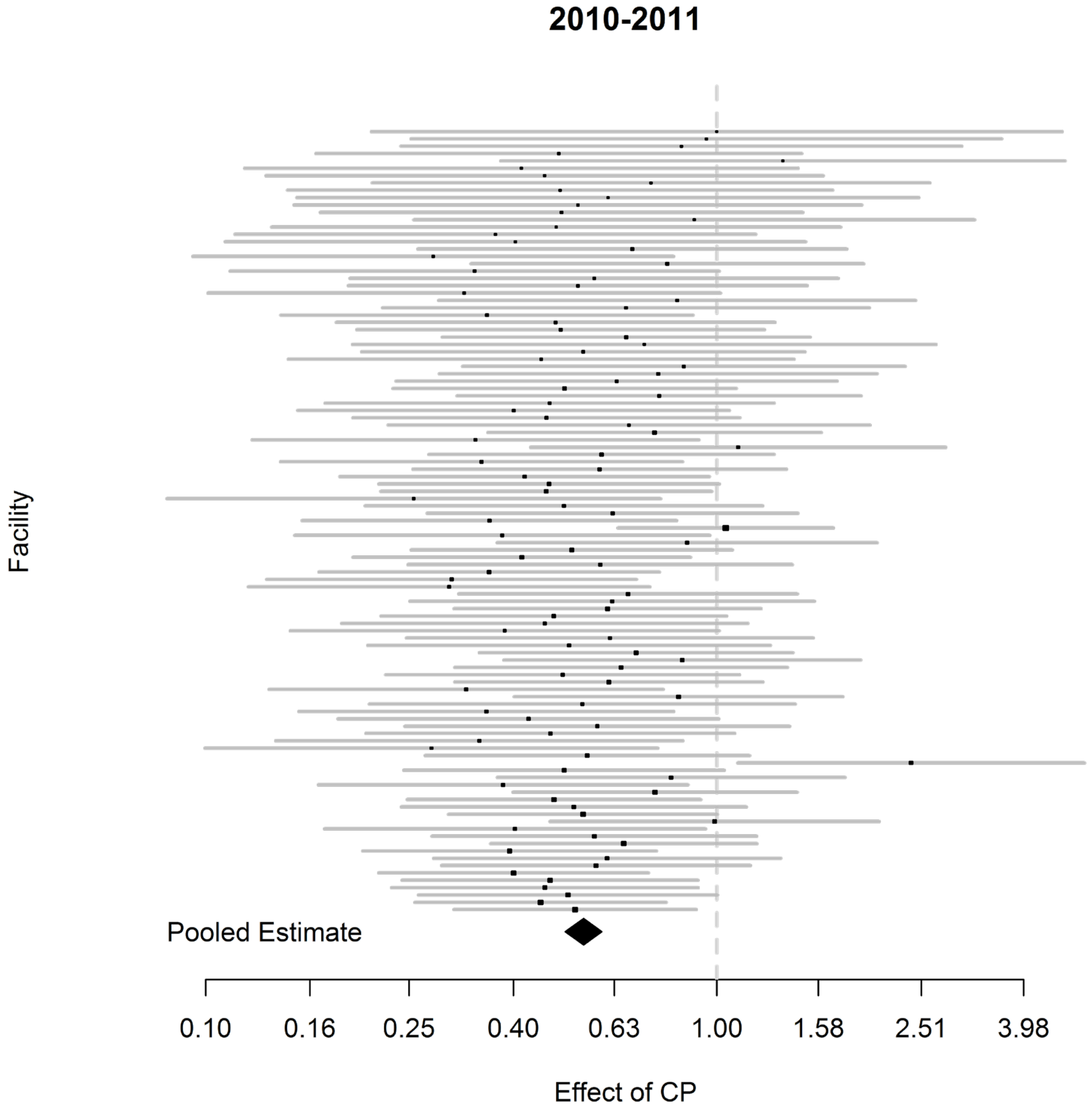

eFigure 3: Forest plot showing the facility-specific estimates along with 95% confidence intervals for each of the facilities during the two years from 2010-2011, and the pooled estimate represented at the bottom.

© 2021 Khader K et al. *JAMA Network Open*.

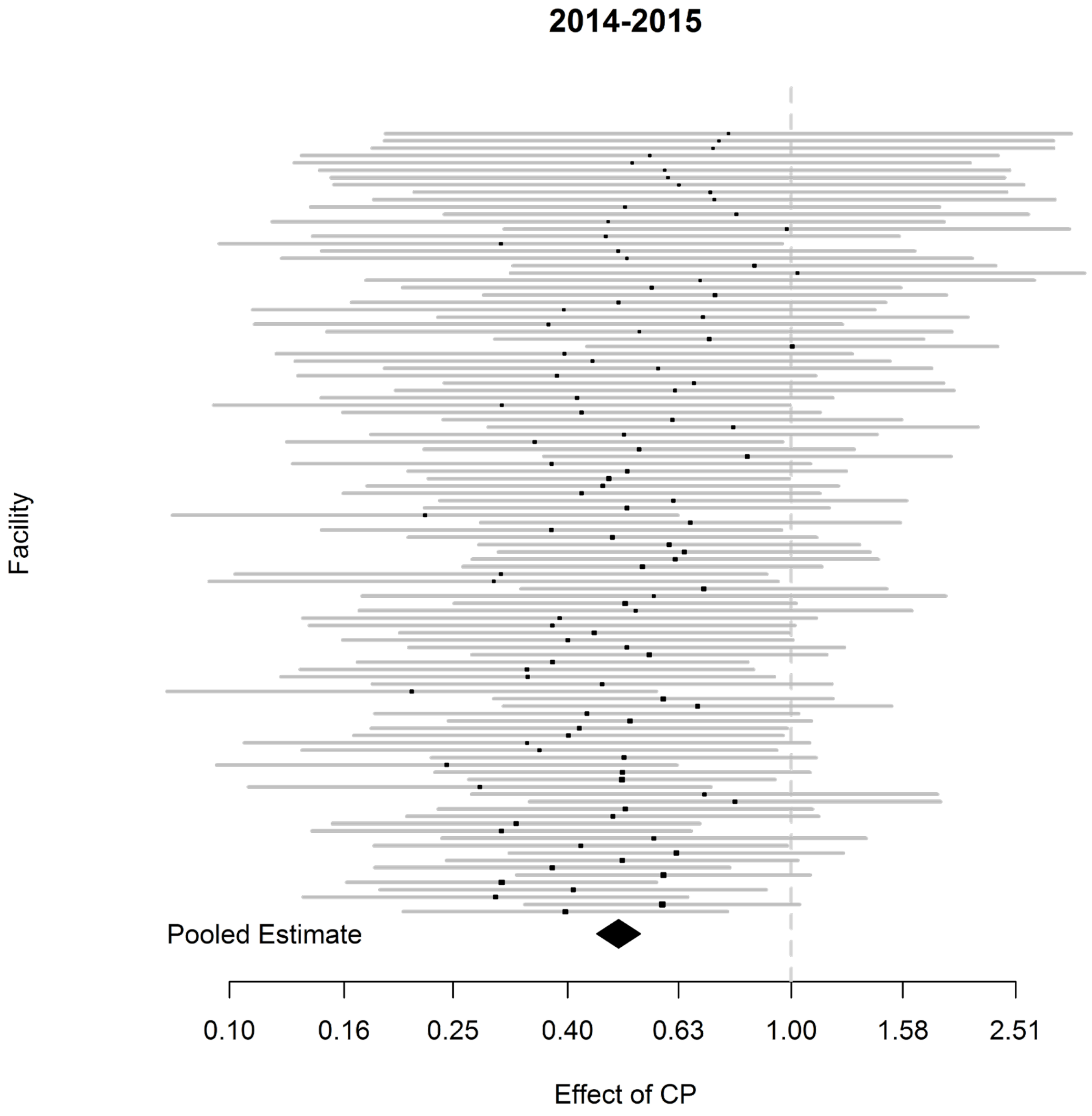

eFigure 4: Forest plot showing the facility-specific estimates along with 95% confidence intervals for each of the facilities during the two years from 2014-2015, and the pooled estimate represented at the bottom.

© 2021 Khader K et al. *JAMA Network Open*.

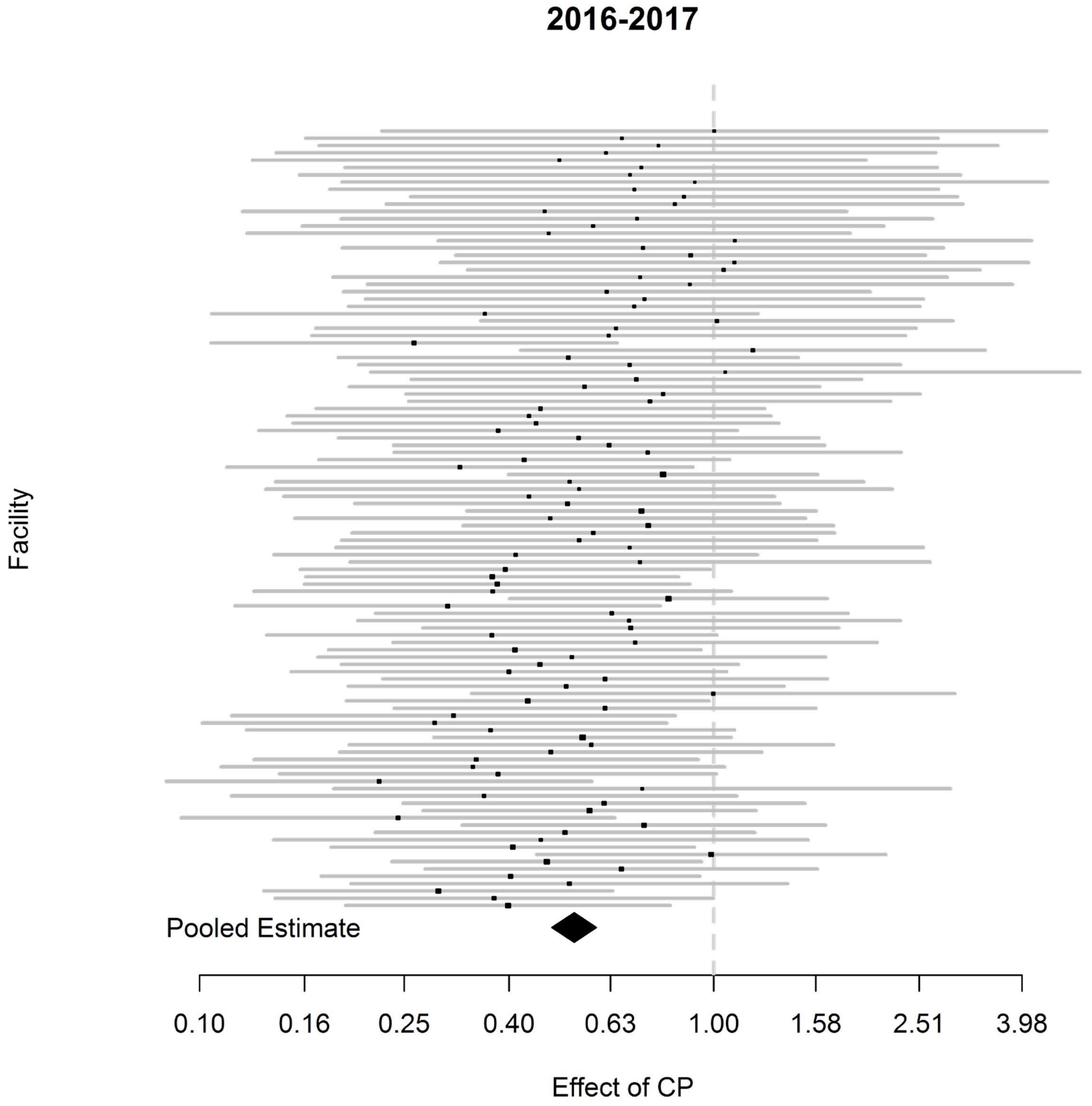

eFigure 5: Forest plot showing the facility-specific estimates along with 95% confidence intervals for each of the facilities during the two years from 2016-2017, and the pooled estimate represented at the bottom.

© 2021 Khader K et al. *JAMA Network Open*.

In the eTable, we summarize the estimates of the model parameters not described in the main text of the manuscript, as these were not of principal focus in this paper. The estimates we obtained here are similar to those reported previously,[6] and were generated by pooling the posterior means across facilities and across time using the R package metafor,[7] while accounting for the facility-specific variability using the posterior standard deviations.

eTable: Estimates of key parameters from the dynamics transmission model along with the 95% confidence intervals (95% CI), pooled across facilities and over time.

PARAMETER	ESTIMATE (95% CI)
**TRANSMISSION RATE (λ)**	0.041 (0.040, 0.042)
**IMPORTATION PROBABILITY (ν)**	0.079 (0.075, 0.083)
**CLEARANCE RATE (η)**	0.020 (0.018, 0.021)
**SURVEILLANCE TEST SENSITIVITY (ϕ)**	0.797 (0.789, 0.806)

The transmission rate in eTable 1 represents the proportional constant that describes how the rate of acquisition changes in relation to the colonization prevalence of MRSA within the wards. This parameter, referred to as the *baseline transmission rate* is the transmission rate not associated with the use of contact precautions, but rather is attributed to patients who are not on contact precautions. The importation probability represents the first-admission importation probability as described above in the eMethods, and is likely lower than the overall importation probability. Indeed during any given time period, admissions may include individuals who were recently hospitalized, and it is plausible that those individuals will be more likely to be MRSA colonized at the time of admission due to their recent exposure to a healthcare setting. The clearance rate of 0.02 corresponds with a mean time until clearance of 50 days (95% CI; 48, 56), which is in line with previous estimates of clearance found in healthcare settings.

eReferences

1. Thomas A, Redd A, Khader K, Leecaster M, Greene T, Samore M. Efficient parameter estimation for models of healthcare-associated pathogen transmission in discrete and continuous time. *Math Med Biol*. 2015;32(1):79-98. doi:10.1093/imammb/dqt021
2. Khader K, Thomas A, Huskins WC, et al. A Dynamic Transmission Model to Evaluate the Effectiveness of Infection Control Strategies. *Open Forum Infect Dis*. 2016;195(1):ofw247. doi:10.1093/ofid/ofw247
3. Khader K, Thomas A, Jones M, Toth D, Stevens V, Samore MH. Variation and trends in transmission dynamics of Methicillin-resistant Staphylococcus aureus in veterans affairs hospitals and nursing homes. *Epidemics*. 2019;28. doi:10.1016/j.epidem.2019.100347
4. Geman S, Geman D. Stochastic Relaxation, Gibbs Distributions, and the Bayesian Restoration of Images. *IEEE Trans Pattern Anal Mach Intell*. 1984;PAMI-6(6):721-741. doi:10.1109/TPAMI.1984.4767596
5. Metropolis N, Rosenbluth AW, Rosenbluth MN, Teller AH, Teller E. Equation of state calculations by fast computing machines. *J Chem Phys*. Published online 1953. doi:10.1063/1.1699114
6. Khader K, Thomas A, Jones M, Toth D, Stevens V, Samore MH. Variation and trends in transmission dynamics of Methicillin-resistant Staphylococcus aureus in veterans affairs hospitals and nursing homes. *Epidemics*. 2019;28. doi:10.1016/j.epidem.2019.100347
7. Viechtbauer W. Conducting meta-analyses in {R} with the {metafor} package. *J Stat Softw*. 2010;36(3):1-48. https://www.jstatsoft.org/v36/i03/

© 2021 Khader K et al. *JAMA Network Open*.
